# Using genome-wide CRISPR library screening with library resistant DCK to find new sources of Ara-C drug resistance in AML

**DOI:** 10.1038/srep36199

**Published:** 2016-11-03

**Authors:** Morito Kurata, Susan K. Rathe, Natashay J. Bailey, Natalie K. Aumann, Justine M. Jones, G. Willemijn Veldhuijzen, Branden S. Moriarity, David A. Largaespada

**Affiliations:** 1 Masonic Cancer Center, University of Minnesota, Minneapolis, MN, USA; 2 Department of Comprehensive Pathology, Graduate School of Medical and Dental Sciences, Tokyo Medical and Dental University, Tokyo, Japan; 3 Department of Pediatrics, University of Minnesota, Minneapolis, MN, USA; 4 Center for Genome Engineering, University of Minnesota, Minneapolis, MN, USA; 5 Brain Tumor Program, University of Minnesota, Minneapolis, MN, USA

## Abstract

Acute myeloid leukemia (AML) can display *de novo* or acquired resistance to cytosine arabinoside (Ara-C), a primary component of induction chemotherapy. To identify genes capable of independently imposing Ara-C resistance, we applied a genome-wide CRISPR library to human U937 cells and exposed to them to Ara-C. Interestingly, all drug resistant clones contained guide RNAs for *DCK*. To avoid *DCK* gene modification, gRNA resistant *DCK* cDNA was created by the introduction of silent mutations. The CRISPR screening was repeated using the gRNA resistant *DCK*, and loss of *SLC29A* was identified as also being capable of conveying Ara-C drug resistance. To determine if loss of *Dck* results in increased sensitivity to other drugs, we conducted a screen of 446 FDA approved drugs using two Dck-defective BXH-2 derived murine AML cell lines and their Ara-C sensitive parental lines. Both cell lines showed an increase in sensitivity to prednisolone. Guide RNA resistant cDNA rescue was a legitimate strategy and multiple *DCK* or *SLC29A* deficient human cell clones were established with one clone becoming prednisolone sensitive. Dck-defective leukemic cells may become prednisolone sensitive indicating prednisolone may be an effective adjuvant therapy in some cases of DCK-negative AML.

Acute myeloid leukemias are myeloid proliferative disease associated with a very poor prognosis in general^1^. In the last 40 years, uncovering genetic abnormalities in leukemia has provided a better understanding of pathogenesis and has helped in the discovery of new disease classifications, prognostic factors and treatments^2^. For example, the targeted therapy Imatinib is effective for treating chronic myelogenous leukemia, however not all leukemias have known molecular targeted therapies and standard chemotherapy including cytarabine (Ara-C) continues to play a core role in the treatment of acute myeloid leukemia (AML). Conventional chemotherapy can currently achieve complete remission in 70–75% of AML cases, however, 60% of these patients eventually relapse after intensive chemotherapies^1^,^3^. At relapse, most patients will no longer respond to Ara-C based induction therapy. Ara-C is a cytidine analog that interferes with DNA replication in fast growing cells and is used in both induction therapy and at relapse. Ara-C is highly effective at eliminating AML blast cells, however, it is typically ineffective in totally eradicating AML. It appears some AML cells are capable of escaping the initial assault by the chemotherapy drugs. We previously described how an *in vitro* model of Ara-C resistance was used to identify one possible explanation for Ara-C resistance, the loss of deoxycytidine kinase (Dck) function^4^. Dck is the rate-limiting enzyme in the metabolic activation of Ara-C. Through the use of *Dck* knockout and rescue experiments, it was shown the loss of *Dck* accounted for over 85% of the Ara-C resistance found in our murine AML cell line B117H^4^.

As cells become resistant to Ara-C, it is likely the cells would become more sensitive to other drugs. Thus, we used standard drug screening to test this theory and identify alternative drugs for Ara-C resistant AML. We interrogated the response of 2 Dck-defective murine cells and their Ara-C sensitive parental lines to 446 FDA approved drugs. The response of the Ara-C resistant cells was compared to the response of their respective parental cells. It was found the Ara-C resistant cells became more sensitive to 3 corticosteroids with the most pronounced change in the glucocorticoid prednisolone. Glucocorticoid prednisolone can induce apoptosis in cells by binding to the glucocorticoid receptor (GR) and continues to play an important role in the treatment of acute lymphoblstic leukemia (ALL) and lymphoid malignancy but not AML^5^.

The CRISPR (clustered regularly interspaced short palindrome repeats) associated nuclease Cas9 system is a new technology that can induce targeted loss-of-function mutations at desired genomic sites by the use of specific guide RNAs^6^. Whole genome CRISPR libraries are powerful tools for genome-scale loss-of-function screening. This system has been previously shown to be highly effective at identifying drug resistant genes *in vitro*. Indeed, Shale *et al*. showed the genes responsible for resistance to the BRAF protein kinase inhibitor vemurafenib (PLX)^7^. We used these CRISPR/Cas9 libraries for exploring Ara-C drug resistance in human AML cell lines to determine which genes are critically important for Ara-C resistance in leukemia relapse. We introduced selective pressure using Ara-C to create drug resistant cells capable of not only resistant to Ara-C but survival and self-renewal to reflect relapse processes.

## Results

### Induction of Ara-C resistant in U937 cells using genome wide CRISPR screening

**Figure 1:**
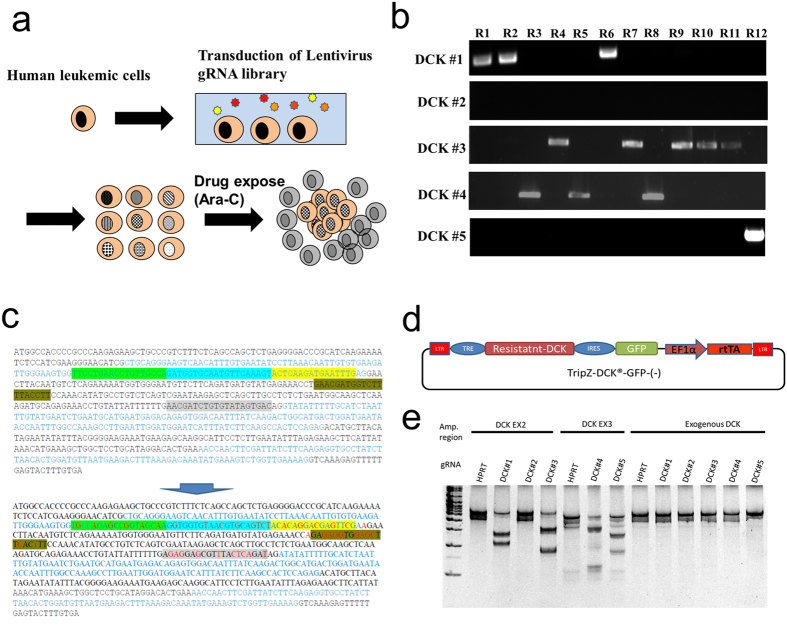
Design for screening and silent mutant of DCK. (**a**) Schema of genome-wide CRISPR library screening. Human leukemic cells were transduced with lentivirus gRNA library, selected in puromysin for 2 weeks and applied to drug selection in 96 well plates. 2~3 weeks later, individual clones were harvested. (**b**) DCK specific PCR for gRNA in library-transduced U937 were treated with 50 μg/ml of Ara-C. DCK#1, DCK#2 and DCK#3 were designed for exon 2 of human *DCK*. DCK#4 and DCK#5 are for exon 3. (**c**) Silent mutations were induced at the highlighted target sites of five gRNA and their PAM sequence. (**d**) Schema of Dox-inducible vector. Transduced cells were sorted by GFP and applied to next experiments. (**e**) The Surveyor assay for the exon2 (Ex2) and the exon 3 (Ex3) region of *DCK* locus and exogenous gRNA resistant *DCK* region. HPRT (hypoxanthine phosphoribosyl transferase 1) was used as a negative control.

Lentiviral particles containing a genome wide set of gRNAs^6^ were used to transduce U937 cells and exposed to puromycin for 2 weeks with minimum passage to select for stable integrants. Cells stably expressing the CRISPR library were then exposed to a high (50 μg/ml) or low (1 μg/m) dose of Ara-C for 2 weeks in 96 well plates and single clones were harvested (Fig. 1a). 360 and 136 clones were identified with low and high dose, respectively, and 12 clones were selected for further investigation from each group to confirm the utility of this library. MOLM13 cells were also transduced with the lentivirus CRISPR library and exposed to Ara-C in the same manner. No clones were observed when cells were exposed to high Ara-C, but 27 clones were observed with low dose Ara-C.

### All Ara-C resistant clones contain gRNAs targeting DCK

Guide RNA regions of each clone were sequenced in the high Ara-C resistant U937 clones (Table 1). All clones were positive for *DCK* gRNA by using gRNA specific PCR (Fig. 1b). In addition, the 12 clones tested from the low dose Ara-C group were also all positive for *DCK* gRNAs by PCR (Fig. S1). This was also observed with the MOLM13 cells transduced with the CRISPR library and selected at low dose Ara-C. However, one clone in the library transduced MOLM13 cells was negative for *DCK* by PCR. Unfortunately, It was determined that this clone had a single nucleotide mutation in the *DCK* gRNA and a mixed mutation was observed in *DCK* exon 2. In total, the CRISPR library contained five different gRNAs for *DCK*: three designed for exon 2 and the other two for exon 3. All U937 clones had a mutation in the *DCK* locus at one of these expected target sites (Table 2).

### Generation of a CRISPR library resistant DCK cDNA expressing cell line

As our screen only identified *DCK* as a candidate Ara-C resistance gene and defective DCK is already a known mechanism of Ara-C resistance^4^,^8^,^9^, we decided to remove the ability of the CRISPR library to knock out *DCK*. To avoid *DCK* gene modification by the CRISPR library, the 5 *DCK* gRNA targeted sites were modified in a cDNA of DCK by introducing silent mutations (Fig. 1c). The CRISPR library resistant *DCK* cDNA was then integrated into U937 cells under the control of a Tet-inducible promoter and transcriptionally linked to GFP (Fig. 1d). GFP-positive cells were isolated and characterized for DCK expression. *DCK* expression was well regulated by doxycycline (Dox), with as little as 1 ng/ml inducing *DCK* expression (Fig. S2). The Surveyor assay was performed and demonstrated the endogenous *DCK* locus was modified by nearly all DCK specific gRNAs in the CRISPR library and, as expected, the exogenous *DCK* cDNA was not modified by any of these gRNA (Fig. 1e).

### CRISPR library screening with library resistant DCK identifies SLC29A as capable of inducing Ara-C resistance

**Figure 2:**
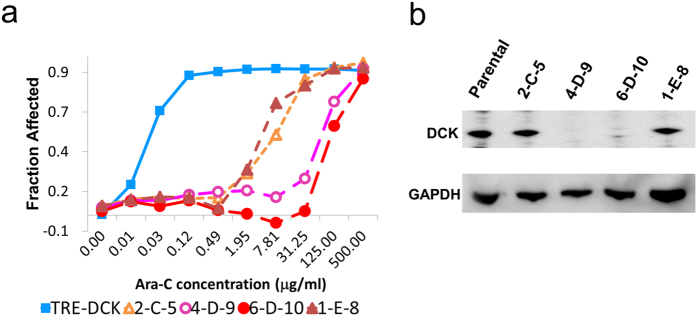
DCK deficient clones were strongly resistant to Ara-C. (**a**) IC50 of each Ara-C resistant clone, as determined by MTS-Tetrazolium assay. 4-D-9 (opened circle) and 6-D-10 (closed circle) had mutations in the *DCK* locus and 2-C-5 (opened triangle) and 1-E-8 (closed triangle) had mutation in *SLC29A* locus. Dox-inducible DCK transduced U937 without doxycycline was used as a control (Closed square). (**b**) Western Blot for DCK expression. Clones 4-D-0 and 6-D-10 were negative for DCK.

The gRNA library was applied to GFP-positive sorted *DCK* inducible U937 cells in the same manner as described with wild type U937 cells. Initially, to establish Ara-C resistant clones, 500 ng/ml of doxycycline was added to induce expression of the exogenous CRISPR resistant DCK and then exposed to 500 ng/ml Ara-C, but no resistant colonies were observed (Table 3). *DCK* expression was well regulated by this concentration of doxycycline (Fig. S2). However, overexpression of DCK may have offset other drug resistant mechanisms in this CRISPR screening. By reducing the concentration of doxycycline to between 1 and 50 ng/ml with 500 ng/ml of Ara-C we were able to successfully obtain Ara-C resistant clones. We analyzed 10 clones from cells treated with 10 ng/ml of Dox and 9 clones from 50 ng/ml of Dox. The gRNA target sites contained in the CRISPR library for *DCK* and *SLC29A* were sequenced and all clones had mutations in either the *DCK* or *SLC29A* locus (Table 4). All clones selected with low dose doxycycline had mutations in *SLC29A* and not *DCK*, which may suggest 50 ng/ml of doxycycline was the optimal concentration for this selection. Established clones were strongly resistant to Ara-C (Fig. 2a) and the clones having a mutation in the *DCK* locus (4-D-9 (IC_50_ = 67.5 μg/ml) and 6-D-10 (IC_50_ = 106.4 μg/ml)) were more resistant to Ara-C than clones having a mutation in *SLC29A* (2-C-5 (IC_50_ = 7.49 μg/ml) and 1-E-8 (IC_50_ = 3.94 μg/ml)). Clone 4-D-9 and 6-D-10 were negative for DCK expression by Western blot analysis (Fig. 2b).

SLC29A is associated with the nucleotide salvage pathway and is required for the uptake and activation of Ara-C. As *DCK* knock-out cells became highly Ara-C resistant we wondered if this loss of function of the nucleotide salvage pathway caused cells to become more susceptible to other types of drugs.

### Ara-C resistant cells lacking functional DCK are sensitive to prednisolone

We conducted a drug screen on four murine BXH-2 derived AML cell lines, two Ara-C resistant (B117H and B140H) and the two Ara-C sensitive parental lines (B117P and B140P), from which the Ara-C resistant cells were derived. These cell lines have previously been reported to contain mutations in *Dck*, causing Ara-C resistance^10^. Four hundred and forty six FDA approved drugs were tested in each of the four cell lines at a concentration of 10 μM, and the results were expressed in the percent of cells inhibited as compared to untreated cells. Of the 446 drugs tested, 165 showed less than a 30% change in the cells’ response to the drug and 218 drugs were inconclusive, as they resulted in killing all of the cells or none of the cells. Forty seven drugs showed a greater than 30% change in only one set of the cells, and 16 showed a greater than 30% change in drug response in both sets of cells (Supplementary Table S1).

Three of the 13 drugs identified by the screen as being more effective at eliminating Ara-C resistant cell lines than parental lines by greater than 30% were classified as corticosteroids (prednisolone, loteprednol etabonate, and corticosterone) (Supplementary Table S2).

### Verification of prednisolone response by determining IC_50_

**Figure 3:**
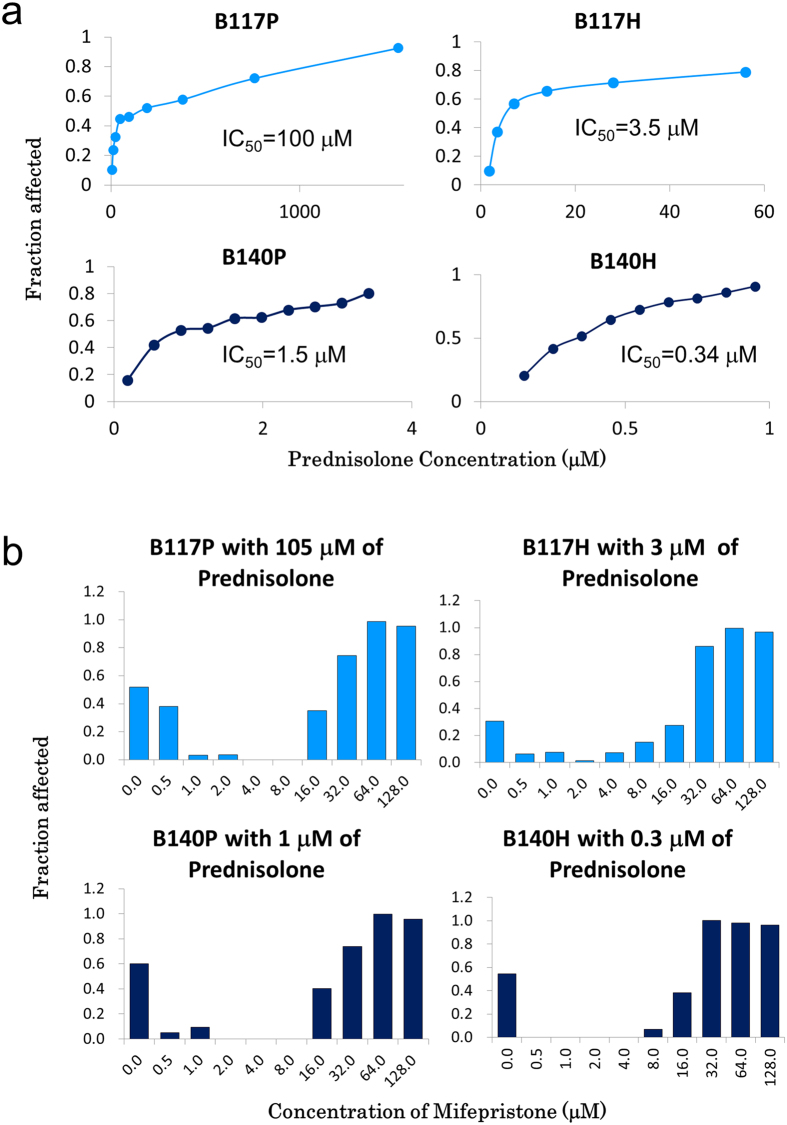
Ara-C resistant murine cells are more sensitive to prednisolone. (**a**) The prednisolone IC_50_ was determined using the MTS Tetrazolium Assay. Assay was repeated 3 times with comparable results. Mifepristone blocks the effects of prednisolone in all four BXH-2 cell lines. (**b**) MTS Tetrazolium Assay with a constant concentration of prednisolone and an increasing concentration of mifepristone. Assay was repeated 3 times with similar results.

Since prednisolone has been used to treat AML, we utilized a MTS tetrazolium assay to establish the IC_50_ for prednisolone (Fig. 3a). The B117P displayed a high level of prednisolone resistance (IC_50_ = 105 mM), but its Ara-C resistant derivative, B117H, had a 20-fold reduction in the prednisolone IC_50_. The B140P cells were even more sensitive to the prednisolone than the B117H cells, and the B140P’s Ara-C resistant derivative, B140H, demonstrated a 3-fold reduction in the prednisolone IC_50_.

Since dexamethasone is a more commonly used glucocorticoid for treating leukemia (clinicaltrials.gov), but was not included in the 446 drugs tested, the IC_50_ for dexamethasone was determined using the MTS Tetrazolium Assay and it provided a similar trend of IC_50_’s in the four cell lines as prednisolone, but at much lower concentrations (Supplementary Table S3).

### Glucocorticoid sensitivity is dependent on binding to the glucocorticoid receptor

Cells respond to glucocorticoids in a number of ways, some of which are independent of glucocorticoid receptor (GR) binding^11^. To determine if the GR was an integral part of the response to prednisolone in the BXH-2 cell lines, a combination drug assay was performed using a constant concentration of prednisolone and a variable concentration of mifepristone. Mifepristone is a steroid antagonist that binds to a number of steroid receptors including the GR^12^. Mifepristone binding to the GR prevents the formation of an activated GR complex even in presence of a GR agonist, such as prednisolone^13^. The drug assay demonstrated mifepristone was able to totally block the toxic effects of prednisolone at concentrations of 2–4 μM in all four BXH-2 cell lines (Fig. 3b). Combination drug assays were also used to determine the combination index CI_50_ for the four cell lines. The resulting CI’s were 5.7, 6.2, 6.0, and 6.6 for the B117P, B117H, B140P, and B140H cells, respectively, all reflective of a high degree of antagonism between prednisolone and mifepristone.

### Ara-C resistant human clones do not always display increased sensitivity to dexamethasone

**Figure 4:**
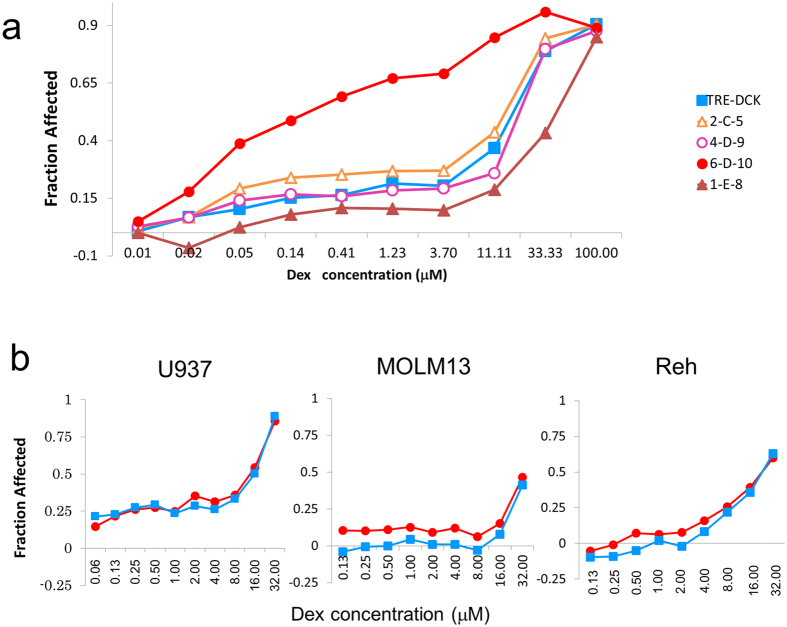
Human Ara-C resistant clone was sensitive to dexamethasone. (**a**) IC_50_ for dexamethasone of each Ara-C resistant clone, as determined by MTS-Tetrazolium assay. 4-D-9 (opened circle) and 6-D-10 (closed circle) had mutations in the *DCK* locus and 2-C-5 (opened triangle) and 1-E-8 (closed triangle) had mutations in the *SLC29A* locus. Dox-inducible DCK transduced U937 without doxycycline was used as a control (Closed square). (**b**) IC_50_ for dexamethasone in U937, MOLM13 and Reh with CRISPR knock-out of *DCK* (closed square), as determined by MTS-Tetrazolium assay. *HPRT (hypoxanthine phosphoribosyl transferase 1*) was used as a negative control (closed circle).

To determine if human AML cells respond in the same way as the murine BXH-2 cell lines, 4 gRNA library resistant U937 clones were tested for glucocorticoid sensitivity. Interestingly, only the 6-D-10 clone (IC_50_ = 0.16 μM) demonstrated increased sensitivity to dexamethasone compare to parental cells (IC_50_ = 15.69 μM) (Fig. 4a). The 6-D-10 clone had a single *DCK* gRNA from the CRISPR library. *DCK* was also knocked-out by CRISPR in U937, Reh and MOLM13 cells and all clones became strongly resistant to Ara-C (Fig. S3a), however, sensitivity to dexamethasone was not changed (Fig. 4b). Loss of DCK expression may increase the chance of development of sensitivity to dexamethasone. To clarify the mechanism of dexamethasone sensitive in human cell lines, the expression of glucocorticoid receptor (*NR3C1*) was measured in U937-CRISPR-library-induced Ara-C resistant clones by the quantitative PCR. However, there were no significant difference among established clones (Fig. S3b). The mechanism for this still remains unknown.

## Discussion

AML is a heterogeneous disease with a diverse collection of mutations driving AML progression in each patient, making it problematic to establish a standard protocol for treating the disease effectively. Moreover, the AML from patients with relapsed/refractory disease have additional therapy-related cellular changes. We sought to clarify which drug resistant mechanism is more dominant in Ara-C resistant. Interestingly, our results strongly indicate the loss of *DCK* is a key factor in Ara-C resistance. In addition, cells harboring a CRISPR resistant *DCK* cDNA circumvented the cells from becoming resistant due to loss of *DCK function* by Cas9 induced mutations. As a result, *SLC29A* was identified as a second gene candidate associated with Ara-C resistance. *DCK* and *SLC29A* are already well known as genes involved in some cases of Ara-C drug resistance^14^,^15^. The downstream kinase required for the conversion of Ara-C monophosphate into Ara-C triphosphate might not be identified in this second CRISPR screen because the downstream kinase, such as CMPK1, is essential for cell survival^16^. Future studies using the CRISPR activation library^17^ may identify other candidates of major deactivating enzymes of the cytarabine metabolic pathway, such as Cytidine deaminase (CDA)^18^ and 5′ nucleotidase (NT5C2)^19^.

Our results directly suggested that loss of DCK provides a better survival advantage than SLC29A in Ara-C resistance, since the IC_50_ of *DCK* deficient cells was more than ten times higher than cells with the loss of *SLC29A* (Fig. 2a). DCK is the rate-limiting enzyme of the salvage pathway and critical for the activation of Ara-C, whereas, the lower IC_50_ for the loss of SLC29A clones suggests SLC29A may not provide the only means for Ara-C’s entrance into cells.

Whole genome CRISPR libraries are powerful tools for genome-scale loss-of-function screens and will certainly reveal mechanisms in various biological phenomena. However, the existence of rate-limiting molecules, like DCK in this study, can prove to be a large stumbling block for whole genome screening. Introduction of CRISPR library resistant cDNAs of these rate-limiting molecules can eliminate redundant gRNA activity and allow for identification of alternative genes involved in the phenotype of interest. To our knowledge, this is the first report describing this type of approach to circumvent identification of dominant rate-limiting molecules.

There are currently over 800 clinical trials underway for treating AML and nearly 1000 completed trials (clinicaltrials.gov). These trials are testing a wide variety of drugs and drug combinations and yet there has been very little improvement in survival trends for AML. Even though over 200 of the active trials include Ara-C in the regime, none evaluate the functional status of deoxycytidine kinase or its pathway members for predicting the effectiveness of Ara-C. Further, the use of synthetic glucocorticoids, such as prednisolone and dexamethasone, in treating AML is very rare. There are only 6 active AML trials using prednisolone and only 5 using dexamethasone (clinicaltrials.gov). These facts underscore the critical need for developing a better understanding of the molecular genetics driving drug response with the goal of providing patient-specific drug options. Perhaps there are some AML patients that would respond to glucocorticoid treatment if the drivers of the glucocorticoid response could be determined. In the case of the BXH-2 and U937 cell lines, the B117P cells are resistant to glucocorticoids, while the B140P and 6-D-10 cells are significantly more sensitive (Figs 3a and 4a). The DCK knock-out with CRISPR in U937 cells did not affect the sensitivity to glucocorticoid. These results suggest glucocorticoid sensitivity may accompany Ara-C resistance under some circumstances, which have yet to be determined.

Synthetic glucocorticoids are given as anti-inflammatory and immunosuppressive agents in the treatment of a variety of ailments, including asthma and graft-versus-host disease^20^,^21^,^22^. Glucocorticoids affect metabolic processes and transcriptional regulation via a number of pathways, most of which are dependent upon binding to the GR^22^. The use of glucocorticoids to treat ALL, but not AML, is due to a mechanism of glucocorticoid-induced apoptosis, which is prevalent in ALL^23^, but not in AML. The drug screen, reported here, evaluating 446 FDA approved drugs indicated that in some cases as cells become resistant to Ara-C they become significantly more sensitive to glucocorticoids, such as prednisolone and dexamethasone. Thus, the response to glucocorticoids in AML cells can be altered by unidentified mechanisms. These results imply there may be some patients with *de novo* AML who might respond well to the inclusion of glucocorticoids in their treatment regimen. To determine when glucocorticoids should be used in the treatment of AML, the functional changes in the cells driving both the Ara-C and the glucocorticoid response need to be identified.

As to the nature of the glucocorticoid response, there are three distinct phases that have been identified in glucocorticoid-induced apoptosis^24^. The first phase, the “initiation stage”, is triggered by glucocorticoid reception binding. The second phase, the “decision stage”, occurs within the mitochondria. The last phase, the “execution stage”, involves the apoptotic caspase cascade. Since all 4 of the BXH-2 cell lines had similar responses to the combination of prednisolone and mifepristone, it appears the glucocorticoid receptor is functioning normally in all 4 lines, and the prednisolone resistance found in the B117P is not related to the “initiation stage”.

The drug assays support the premise that the BXH-2 cell lines are useful tools for understanding the molecular characteristics driving drug response. For example, the assay combining a fixed amount of prednisolone with variable amounts of mifepristone confirmed a high degree of antagonism between the drugs in all four cell lines confirming the glucocorticoid apoptotic activity in leukemia cells is dependent on glucocorticoid reception binding.

For this present study, a genetic screening and a drug screen were performed. The CRISPR screening showed normal DCK and SLC29A function is critical for Ara-C sensitivity in human AML cell lines. The drug screening of 446 single drugs determined AML cells became more sensitive to other drugs when they became resistant to Ara-C (a model of refractory AML). The drug screen showed the BXH-2 cells became more sensitive to glucocorticoids as they became resistant to Ara-C. Uncovering the significant cellular characteristics associated with these responses, such as gene expression levels or mutated genes, could be instrumental in developing tests to determine the most effective drug combinations for treating each AML patient.

## Methods

### Cell culture and lentivirus transduction

U937, a human leukemic monocyte lymphoma cell line, MOLM13, a human acute myeloid leukemia cell line and Reh, a human acute lymphocytic leukemia (nonT; nonB) cell line, were maintained in RPMI 1640 medium supplemented with 10% (v/v) fetal bovine serum (FBS). The B117P, B117H, B140P, and B140H (murine BXH-2) cell lines were maintained at 37 °C in 10% CO2 in ASM^4^.

To produce lentivirus, a total of 6 × 10^6^ HEK293T cells were seeded in a 6 well plate, the day before transfection in DMEM medium with 10% fetal bovine serum. Transfection was performed using Lipofectamine 2000 (Thermo Fisher Scientific, Waltham, MA). 18 μg of lentivirus plasmid, 6 μg of pMD.G-VSV and 12 μg of pCMVDR8.2 were diluted in 900 μl of OptiMEM and 60 μl of Lipofectamine 2000 was diluted in 900 μl of OptiMEM then added to the mixture of DNA reagent. The complete mixture was incubated for 15 min before being added to cells. After 16 h, the media was changed to new RPMI 1640 medium supplemented with 10% fetal bovine serum. After 48 h, the media was removed and centrifuged at 1,500 rpm at 4 °C for 10 min to pellet cell debris. Cells were transduced with the Lentivirus via spinfection supplemented with 5 μg/ml polybrene (Sigma-Aldrich, St. Louis, MO). Lentivirus titer was measured with qPCR Lentivirus Titration Kit (Applied Biological Materials Inc. CANADA). Multiplicity of infection (M.O.I.) was calculated and is provided a range between 1.9 and 8.5, which could result in single clones having multiple gRNAs.

### CRISPR library screening

Lenti-virus CRISPR plasmid and CRISPR Library Version One were purchased form Addgene (Cambridge, MA). To identify the relapse responsible gene, the following model was designed (Fig. 1a). U937 cells (3 × 10^7^ cells) were transduced with GeCKO CRISPR Library Version One and then treated with Puromycin for 2 weeks and then 5 × 10^4^ cells were seeded per well of each 96 well plate and six 96 well plate were exposed to Ara-C (Sigma-Aldrich, St. Louis, MO). After 2–3 weeks, Ara-C resistant clones were harvested individually. To identify which gRNA was transduced, the region containing gRNA were amplified by the following primers with using the AccuPrime™ *Taq* DNA Polymerase, high fidelity (Thermo Fisher Scientific, Waltham, MA). GeCKO 1717F 5′-gagggcctatttcccatgat-3′ and GeCKO 3913R 5′-cggtgccactttttcaagtt-3′. Genomic DNA isolations from each of the cell lines were performed using a DNeasy Blood & Tissue Kit (QIAGEN). The resulting samples were quantified using a NanoDrop^TM^ 1000 Spectrometer (Thermo Fisher Scientific Inc.). DNA samples were stored at −20 °C. The fragments were sequenced by TA-cloning with pGEM **-**T Vector Systems (Promega, Madison, WI). GeCKO library contains five different gRNAs for *DCK*: #1; actttgaacattgcaccatc, #2; ttcctgaacctgttgccaga, #3; aagtactcaagatgaatttg, #4; aaggtaaaagaccatcgttc, #5; aacgatctgtgtatagtgac. #1, #2 and #3 were designed in Exon2 of *DCK* and #4 and #5 in Exon3. To amplify genomic DNA region of *DCK*, g*DCK* Ex2 F; 5′-gcagggagccttttcatttt-3′ and g*DCK* Ex2 R; 5′-ccattgatatggagagccaac-3′ were used for Exon2, g*DCK* Ex3 F; 5′-gccctattgaccattaattttgc-3′ and g*DCK* Ex3 R; 5′-accagactgctgagggattt-3′ were used for Exon3.

### Inducible gRNA-resistant DCK overexpression vector

gRNA-resistant-silent-mutated cDNA of *DCK* (Fig. 1c) with Attb sites was purchased from G-block (Integrated DNA Technologies, Inc.). The mutant *DCK* cDNA was then transferred to the pENTR vector via the Invitrogen^TM^ Gateway^®^ BP Clonase^®^ reaction and then transferred to TripZ-TRE-DEST-IRES-GFP-EF1A-rtTA via the Invitrogen^TM^ Gateway^®^ LR Clonase^®^ reaction (Thermo Fisher Scientific, Waltham, MA), following manufacturer’s instructions. TripZ-TRE-*Dck*-IRES-GFP-EF1A-rtTA transduced and exposed to 1.0 μg /ml of doxycycline and GFP positive cells were sorted with AriaΙΙ (BD Biosciences).

### Drug assays

Drug assays were performed using the CellTiter 96^®^ Aqueous Non-Radioactive Cell Proliferation Assay (Promega, Madison, WI), as described previously^4^. For each cell line the drug was tested using 10 different concentrations, and each drug concentration was tested in quadruplicate. The drug concentrations were selected to maximize the number of data points between IC_5_ and IC_95_. For the results to be acceptable there needed to be data points on both sides of the IC_50_ and the *r*-value needed to be greater than 0.85. Inhibitory concentrations (IC) values and *r*-values were calculated using CalcuSyn 2.0 (Biosoft, Cambridge, UK).

### Screen of 446 FDA approved drugs

A screen using a NIH Clinical Collection of 446 FDA approved drugs (http://www.nihclinicalcollection.com/) at 10 μM was conducted for each of the four BXH-2 cell lines (B117P, B117H, B140P and B140H). Cell viability was measured using Alamar Blue and fluorescence readout on a Molecular Devices Spectromax 2e microplate reader.

### Western blot analysis

Whole cell lysates were separated on a NuPAGE^®^ Novex^®^ 10% Bis-Tris Gel (Life Technologies Corporation) and transferred to PVDF membrane. The membrane was blocked with Tris-buffered saline (pH 7.5) containing 0.2% Tween 20 and 5% nonfat dry milk. Primary antibodies used were as follows: rabbit anti-DCK (GeneTex, Inc. CA) and anti-Gapdh (1:10,000, Cell Signaling Technology, Danvers, MA). Goat anti-Rabbit HRP conjugated secondary antibodies were utilized at 1:5000 dilution (Santa Cruz Biotechnologies, Dallas, TX). Membranes were developed with Western Bright Ecl kit (BioExpress, Kaysville, UT).

## Additional Information

**How to cite this article**: Kurata, M. *et al*. Using genome-wide CRISPR library screening with library resistant DCK to find new sources of Ara-C drug resistance in AML. *Sci. Rep.* **6**, 36199; doi: 10.1038/srep36199 (2016).

**Publisher’s note:** Springer Nature remains neutral with regard to jurisdictional claims in published maps and institutional affiliations.

## Supplementary Material

Supplementary Information

## Figures and Tables

**Table 1:** Analysis of gRNAs sequence in Ara-C resistant clones.

Clone No.	TA- No.	Sequence of gRNA	CCDS ID in library	Gene name	Exon No.
R2	2-1	CAGCGCCACCAATTCCAACC	Not matched		
	2-2	CTCCTTATGGGCATGAGTGA	Not matched		
R3	3-1	AAGGTAAAAGACCATCGTTC	CCDS3548.1	*/**DCK***	3
	3-2	AAGGTAAAAGACCATCGTTC	CCDS3548.1	*/**DCK***	3
	3-3	AAGGTAAAAGACCATCGTTC	CCDS3548.1	*/**DCK***	3
	3-4	AAGGTAAAAGACCATCGTTC	CCDS3548.1	*/**DCK***	3
	3-5	AAGGTAAAAGACCATCGTTC	CCDS3548.1	*/**DCK***	3
R4	4-1	TGGCGCGGGACACGCGGCAAG	Not matched		
R5	1-1	AAGGTAAAAGACCATCGTTC	CCDS3548.1	*/**DCK***	3
R6	6-1	ACGCTCCTTGAGCCACAGTA	CCDS58992.1	*/OR2V1*	1
	6-2	ACGCTCCTTGAGCCACAGTA	CCDS58992.1	*/OR2V1*	1
	6-3	AGTCCCTGCGCGAGCACTA	Not matched		
	6-4	ACTTTGAACATTGCACCATC	CCDS3548.1	*/**DCK***	2
R7	7-1	CCAGTCAA	Not matched		
	7-2	TTCGTTGCCCTCTGTGCCAT	Not matched		
	7-3	CAGACGCTTCCAGTCTATGC	CCDS13065.1	*/CDC25B*	5
	7-4	GTCAAGTTTCGCTGCCCAGC	CCDS31298.1	*/FGFR2*	4
	7-5	CAGACGCTTCCAGTCTATGC	CCDS13065.1	*/CDC25B*	5
R8	8-2	GATTGTGACTCCATTTGCTC	CCDS47213.1	*/PDE4D*	3
	8-4	ACCACCTAGTATTGCTACCA	CCDS58074.1	*/CREM*	3
	8-5	ACCACCTAGTATTGCTACCA	CCDS58074.1	*/CREM*	3
	8-6	AAGGTAAAAGACCATCGTTC	CCDS3548.1	*/**DCK***	3
	8-7	GATTGTGACTCCATTTGCTC	CCDS47213.1	*/PDE4D*	3
R9	9-2	AAGTACTCAAGATGAATTTG	CCDS3548.1	*/**DCK***	2
R10	10-1	CAGCACCGCCGATGGCCGCT	CCDS5060.2	*/SCML4*	3
	10-2	CCCATGTTCCTCTTCACAG	Not matched		
R11	11-1	TTTCCTAGCAATAGGCGTTT	Not matched		
	11-2	AAGTACTCAAGATGAATTTG	CCDS3548.1	*/**DCK***	2
	11-3	GGTCGGGCAGACCTTATTGC	Not matched		

**Table 2:** Analysis of *DCK* locus mutation.

Clone number	Ex2	Ex3
R1	Mixed mutation	No
R2	Deletion	No
R3	Deletion	No
R4	No	Mixed mutation
R5	No	Mixed mutation
R6	No	Insertion
R7	No	Deletion
R8	No	Mixed mutation
R9	Deletion	No
R10	No	Mixed mutation
R11	No	Mixed mutation
R12	Deletion	No

**Table 3:** The number of drug resistant clones in each experiment.

Cells	Ara-C (ng/ml)	Dox (ng/ml)	Colony number/10^7^ cells
U937	50000	0	41.85
	1000	0	110.77
U937-TRE-Resistant DCK	1000	500	0.00, 0.00
	500	500	0.00
	1000	0	8.00
	500	0	1.00, 5.00, 5.26, 7.89
	500	50	16.07
	500	10	5.33, 6.67
	500	1	2.50

**Table 4:** Analysis of *DCK* and *SLC29A* locus mutation in Ara-C resistant U937-mutant DCK expressing cells.

Dox Conc.	Clone number	*DCK* Ex2	*DCK* Ex3	*SLC29A*
10 ng/ml	2-C-3	**Yes**	No	No
	2-C-5	No	No	**Yes**
	3-E-7	No	No	**Yes**
	4-C-9	**Yes**	No	No
	4-D-9	**Yes**	No	No
	4-E-9	No	No	**Yes**
	5-D-9	No	No	**Yes**
	6-C-9	No	No	**Yes**
	6-E-5	**Yes**	No	No
	6-D-10	No	**Yes**	No
50 ng/ml	1-C-5	No	No	**Yes**
	1-E-3	No	No	**Yes**
	1-E-8	No	No	**Yes**
	2-E-4	No	No	**Yes**
	2-C-8	No	No	**Yes**
	3-D-3	No	No	**Yes**
	3-D-5	No	No	**Yes**
	3-E-8	No	No	**Yes**
	2-D-4	No	No	**Yes**
