# Supplementary material for: Using genome-wide CRISPR library screening with library resistant DCK to find new sources of Ara-C drug resistance in AML
Source: Sci Rep. 2016 Nov 3;6:36199. doi: 10.1038/srep36199 (PMC5093682; doi:10.1038/srep36199)
Supplement: Supplementary Information [file srep36199-s1.pdf]

Using genome-wide CRISPR library screening with library resistant DCK to find new sources of Ara-C drug resistance in AML

Morito Kurata[1,2,*], Susan K. Rathe[1], Natashay J. Bailey[1], Natalie K. Aumann[1], Justine M. Jones[1], G. Willemijn Veldhuijzen[1], Branden S. Moriarity[1,3,4], David A. Largaespada[1,3,4,5]

Supplementary Figures and Tables

Supplementary Fig. S1 Kurata et al

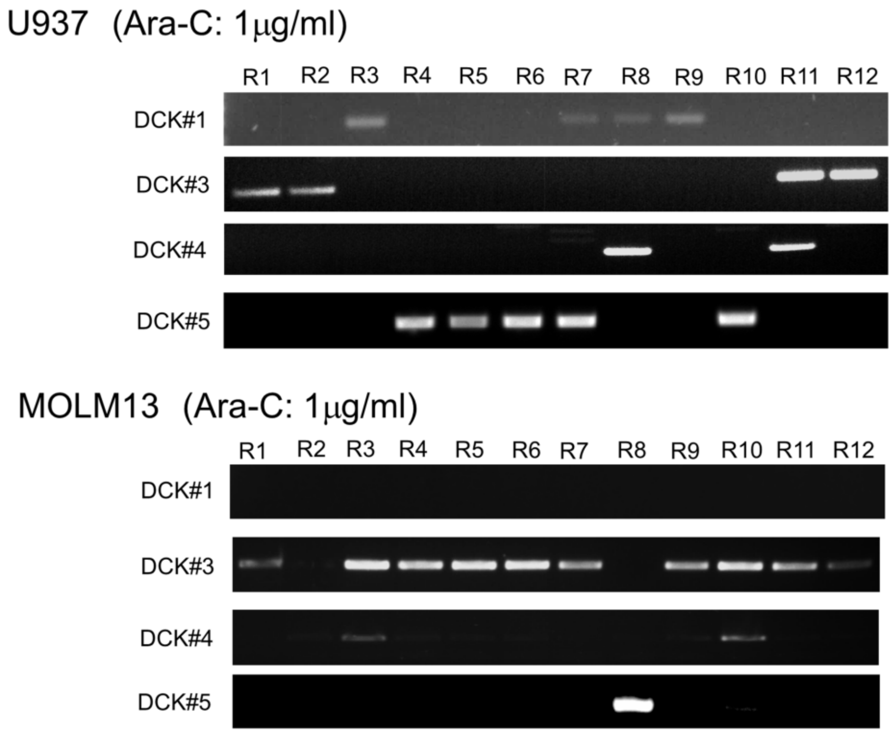

Supplementary Fig. S2 Kurata et al

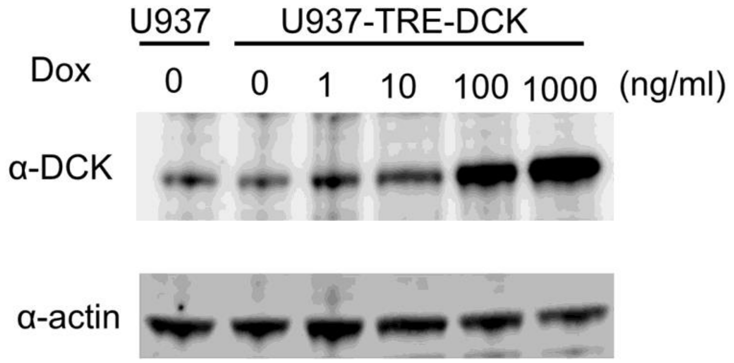

Supplementary Fig. S3 Kurata et al

a

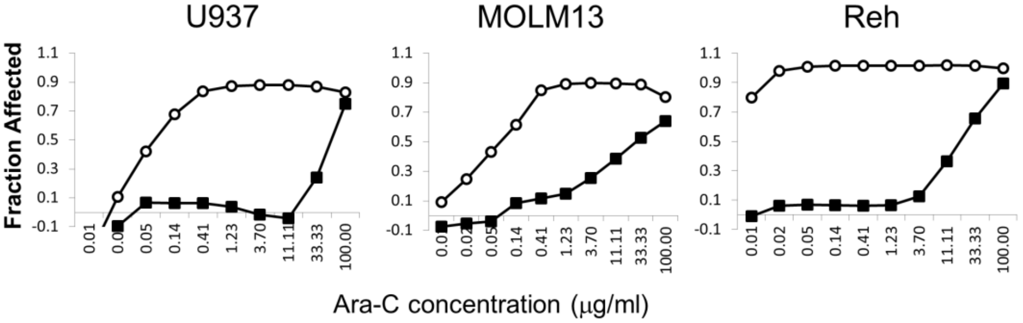

b

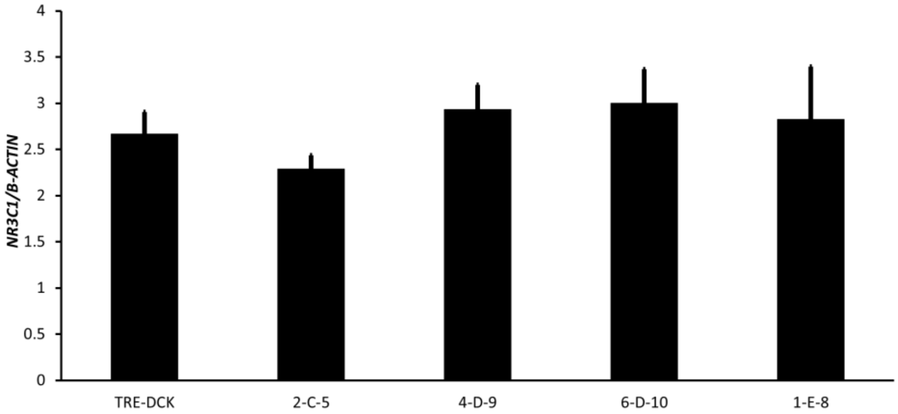

Supplementary Fig. S1: gRNA for DCK specific PCR

DCK specific PCR in library-transduced U937 and MOLM13 cells selected with 1 μg/ml of Ara-C.

Supplementary Fig. S2: Concentration depending expression of silent mutant DCK (A) Protein expression of DCK in doxycycline-inducible DCK transduced U937 cells with different concentration of doxycycline.

Supplementary Fig. S3: CRISPR knock-out of DCK and Quantitative PCR for NR3C1 in Ara-C resistant clones.

(a) IC_{50} for Ara-C in U937, MOLM13 and Reh with CRISPR knock-out of *DCK* (closed square), as determined by MTS-Tetrazolium assay. *HPRT (hypoxanthine phosphoribosyl transferase 1) was used as a negative control* (opened circle).

(b) Quantitative-PCR for glucocorticoids receptor (*NR3C1*) was performed with 5'-tgccagagttgatattcactgat-3' and 5'- tccatcacatctcccctctc-3' among Ara-C resistant clones.

Supplementary Table S1: Summary of Drug Screen of 446 FDA Approved Drugs

446 FDA approved drugs were tested in the B117P, B117H, B140P and B140H cell lines. Each drug was tested at concentration of 10μM. The percent of effect for each of the drugs tested were summarized and categorized.

**Response of Cells to Drugs in Drug Screen**	**Drug Count**
B117H and B140H cells became more sensitive than parentals by > 30%	13
B117H and B140H cells became more resistant than parentals by > 30%	3
B117H became more sensitive than B117P by > 30%	15
B117H became more resistant than B117P by > 30%	2
B140H became more sensitive than B140H by > 30%	29
B140H became more resistant than B140H by > 30%	1
Less than 30% change in both sets of cells	165
All 4 cell lines resistant to drugs (< 10% inhibition)	198
All 4 cell lines sensitive to drugs (> 90% inhibition)	20
**Total**	**446**

Supplementary Table S2: 13 Drugs Have a Higher Effect in Ara-C Resistant Cells

13 of the drugs tested in the B117P, B117H, B140P and B140H cell lines had an increase in percent affected of >30% in the Ara-C resistant cell lines (B117H and B140H).

			% affect of 10μM on each cell line				Least
**Compound**	**Description**	**Type**	**B117P**	**B117H**	**B140P**	**B140H**	**change**
SAM001246965	AM404	Analgesic	48.3	95.5	-6.7	52.9	47.2
SAM001246897	Prednisolone	Corticosteroid	29.2	72.3	34.7	79.1	43.1
SAM001246630	Loteprednol etabonate	Corticosteroid	34.9	74.7	22.9	70.8	39.8
SAM001247051	Mesoridazine	Phenothiazine	9.1	47.8	-1.7	58.4	38.7
SAM001247098	R(+)-SCH-23390 hydrochloride	D_1 receptor antagonist	2.7	38.5	23.7	87.4	35.8
SAM001247066	CGS 12066B	5-HT_{1b} agonist	12.4	47.8	-3.0	58.2	35.4
SAM001246750	Aripiprazole	Antipsychotic	-13.9	49.6	3.6	38.4	34.8
SAM001247070	Pergolide mesylate salt	Dopaminergic agonist	-2.9	49.0	-8.6	25.4	34.0
SAM001246879	Corticosterone	Corticosteroid	24.2	57.3	6.7	53.1	33.1
SAM001246658	Naftopidil	α1-adrenergic receptor antagonist	-25.6	48.1	-2.1	30.7	32.8

Supplementary Table S3: IC_{50}'s for Prednisolone and Dexamethasone in BXH-2 Cell Lines

The IC_{50} for prednisolone and dexamethasone was determined using the MTS Tetrazolium Assay. Assay was repeated a minimum of 3 times. r-values > 0.88.

		Cell Line			
**Drug**	**Statistic**	**B117P**	**B117H**	**B140P**	**B140H**
Prednisolone	Average IC50 (μM)	100.198	3.450	1.547	0.338
	Standard Deviation	14.348	0.980	0.952	0.072
Dexamethasone	Average IC50 (μM)	1.468	0.183	0.125	0.032
	Standard Deviation	0.393	0.047	0.061	0.006
